# Laser Ablation Mechanism and Performance of Carbon Fiber-Reinforced Poly Aryl Ether Ketone (PAEK) Composites

**DOI:** 10.3390/polym14132676

**Published:** 2022-06-30

**Authors:** Jindong Zhang, Ran Bi, Shengda Jiang, Zihao Wen, Chuyang Luo, Jianan Yao, Gang Liu, Chunhai Chen, Ming Wang

**Affiliations:** 1 Center for Advanced Low-Dimension Materials, State Key Laboratory for Modification of Chemical Fibers and Polymer Materials, College of Materials Science and Engineering, Donghua University, Shanghai 201620, China; zhangjindong@buaa.edu.cn (J.Z.); yjn@dhu.edu.cn (J.Y.); cch@dhu.edu.cn (C.C.); mwang@dhu.edu.cn (M.W.); 2 Shanghai High Performance Fibers and Composites Center (Province-Ministry Joint), Center for Civil Aviation Composites, Donghua University, Shanghai 201620, China; 2210510@mail.dhu.edu.cn (R.B.); jiangshengda321@163.com (S.J.); 15221820839@163.com (Z.W.)

**Keywords:** thermoplastic composites, ablation mechanism, continuous wave laser

## Abstract

The ablation mechanism and performance of carbon fiber (CF)-reinforced poly aryl ether ketone (PAEK) thermoplastic composites were studied in this paper. The results show that the ablation damaged area is controlled by the irradiation energy, while the mass loss rate is controlled by the irradiation power density. In the ablation center, the PAEK resin and CFs underwent decomposition and sublimation in an anaerobic environment. In the transition zone, the resin experienced decomposition and remelting in an aerobic environment, and massive char leaves were present in the cross section. In the heat-affected zone, only remelting of the resin was observed. The fusion and decomposition of the resin caused delamination and pores in the composites. Moreover, oxygen appeared crucial to the ablation morphology of CFs. In an aerobic environment, a regular cross section formed, while in an anaerobic environment, a cortex–core structure formed. The cortex–core structure of CF inside the ablation pit was caused by the inhomogeneity of fibers along the radial direction and the residual carbon layer generated by resin decomposition in an anoxic environment. The description of the ablation mechanism presented in this study broadens our understanding of damage evolution in thermoplastic composites subjected to high-energy CW laser irradiation.

## 1. Introduction

Carbon fiber (CF)-reinforced polymer composites (CFRPCs) have become important structural materials in the aeronautic industry because of their outstanding properties, such as high specific stiffness, strength-to-weight ratio, and low thermal expansion coefficient [1,2,3]. Recently, the rapid development of high-energy laser weapons has become a new kind of threat to CFRPC applications in aircraft [4,5]. Generally, structural damage induced by lasers is attributed to the thermal ablation effect. The high-energy laser can cause resin decomposition, fiber fracture, delamination, and even direct penetration. This damage will seriously reduce the bearing capacity of composites and threaten the safety of internal electronic components and equipment. Therefore, it is of great significance to investigate the damage process and ablation mechanism of CFRPCs subjected to laser irradiation, which is prospective for the evaluation of residual strength and laser protection of composites applied in aircraft [6].

Currently, research on laser damage to resin matrix composites mainly focuses on the physical damage and ablation behavior of the material. At the physical level, it was found that laser shock can cause composite delamination. Regarding the ablation behavior, researchers analyzed the decomposition and sublimation reactions that occurred in the composites and discussed the influencing factors. Ecault et al. [7] quantified the residual deformation of composites after laser damage by optical microscopy, X-ray radiography, and interferometric confocal microscopy. They successfully measured the defect position and deformation morphology of T800/M21 composites under different power densities of the laser shock. Ferrante et al. [8] investigated the effect of basalt fiber hybridization on the damage tolerance of carbon/epoxy laminates subjected to laser shock waves and found that such sandwich structures had excellent damage tolerance. Kaludjerovi et al. [9] studied the ablation behavior of carbon/epoxy composites under pulsed-wave (PW) laser ablation at different energy densities. The depth and area of the crater-like ablation zone were quantitatively analyzed by ImageJ software, and the ablation area curve under different energy densities was fit. The results showed that the area of the ablation zone was positively related to the laser energy density. Multidirectional composites showed a higher damage threshold than unidirectional composites because of their uniform thermal conductivity. Ma et al. [10] analyzed the ablation behavior of glass/polybenzoxazole composites under continuous-wave (CW) laser irradiation and characterized the morphology as well as measured the mass ablation rate after ablation under different power densities. It was found that laser parameters such as power density and irradiation time have significant effects on the damage of composite materials. A mixture of residual coke and molten glass fiber can cover the surface of the ablation pit to prevent oxidation. Compared with experiments, finite element analysis (FEA) offers an effective approach to simulate temperature field, stress field, and damage effect of resin matrix composites. Gay et al. [11] studied the delamination damage of laminates ablated by PW lasers at different power densities and obtained the interlaminar stress through finite element simulation. They proved that the laser shock generated axial tension on the composite laminates, thereby causing delamination. Aiming at determining the thermomechanical properties of composites, Liu et al. [12] investigated the effect of CW laser ablation on the interlaminar damage of T700/BA9916 composites under different laser parameters and analyzed the interface stress by thermomechanical coupling simulation. It was found that interlaminar cracks caused by maximum normal tensile stress and interlaminar shear stresses appeared in the cooling stage, when the maximum temperature gradient was increased in the laminates. Based on the bridging theory, Liu et al. [13] proposed a multiscale progressive damage model of carbon/epoxy composites under laser mechanical coupling, which can effectively predict the failure load of laminates. Nan et al. [14] studied the ablation process of composite laminates under CW laser irradiation through tests and simulations. They found that the difference in thermal conductivity of material components and the layup scheme of laminates have a great effect on the ablation morphology. Sihn et al. [15] established a prediction model for the temperature distribution of T650/bismaleimide composites heated by a laser and adopted an infrared thermal imager to verify the temperature. The nonlinear transient 2D finite element model can precisely predict the temperature distribution of specimens at both the heating and the cooling stages. The variety of environmental conditions also has an incredible influence on the ablation behavior of resin matrix composites. Sato et al. [16] compared and analyzed the influence of different types of airflow, such as atmosphere, dry air, and nitrogen, on the laser cutting of laminates. They found that the temperature and the area of the heat-affected zone dramatically decreased in dry air and nitrogen environments. The above research comprehensively discussed the decomposition and sublimation reactions in resin matrix composites during laser irradiation and the delamination phenomenon of composites under laser shock. Additionally, further discussion on the laser irradiation effects was conducted by considering the material composition, microstructure, blowing airflow, etc.

In summary, the ablation mechanism of CFRPCs under laser irradiation has been extensively studied in experiments and simulations. However, the above literature was primarily focused on traditional thermoset matrix composites. With the gradual improvement of the properties of high-performance thermoplastic (TP) matrix materials, thermoplastic composites (TPCs) are expected to be widely used in next-generation aircraft, owing to their high toughness, outstanding damage tolerance, and recoverability [17,18,19,20,21]. The thermophysical properties of TP resin are quite different from those of thermosetting resin. Thermosetting resins form an infusible cross-linked network structure after curing. Therefore, they only decompose at high temperature [22], while thermoplastic resins experience remelting and decomposition [23]. Thus, the laser ablation mechanism of TPCs is also different at certain temperatures [24,25], which will result in different damage modes and degrees of composite structures. Hence, it is necessary to study the ablation mechanism of TPCs. From the above review, however, it appears that there is little work focused on this subject. Compared with PW lasers, CW lasers are closer to the damage mode of laser weapons, and irradiation can make the temperature of an object rise sharply to hundreds or even thousands of degrees Celsius, which is commonly used in extreme temperature tests and finish machining of materials [26]. Moreover, it is also an effective method to simulate the damage of structural materials by laser weapons. Therefore, the present work concentrates on the ablation mechanism and performance of CF-reinforced thermoplastic composites (CFRTPCs) under CW laser irradiation. First, CFRTPCs were prepared by a hot-pressing process. Second, CW laser ablation tests were carried out to investigate the ablation performance of the composites by changing the laser power, irradiation time and spot diameter. Third, the macromorphology and microstructure of the ablated CFRTPCs were characterized. Finally, the microablation evolution behavior was deduced, and the ablation mechanism was proposed. This work contributes to the damage assessment of CFRTPCs subjected to CW laser irradiation.

## 2. Experiment

### 2.1. Materials and Specimens

**Figure 1:**
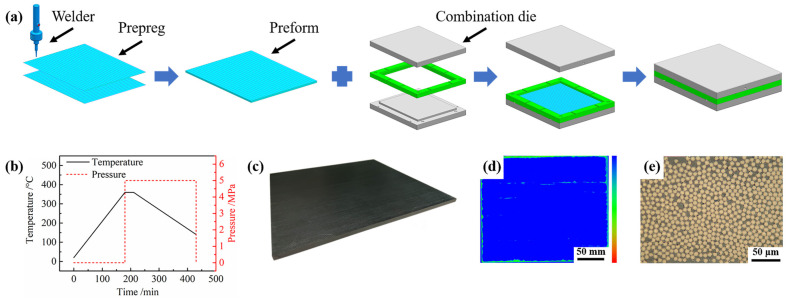
Preparation process and morphology of CF/PAEK: (**a**) schematic illustration of the preparation process; (**b**) curing cycle; (**c**) CF/PAEK after curing; (**d**) ultrasonic C-scan result; (**e**) optical microscope photo of the cross section.

The CFRTPCs were prepared from CF (TZ700S-12K, Weihai Tuozhan Fibre Co., Ltd. Weihai, China)-reinforced poly(aryl ether ketone) (PAEK-L) unidirectional prepreg provided by Heilongjiang Yingchuang New Materials Co., Ltd. Jiamusi, China The areal density of the CF was 149 g/m^2^, and the resin content of the prepreg was 37 wt%. Moreover, the nominal ply thickness of the prepreg was 0.15 mm. The thermophysical properties of the CF and PAEK-L resin are presented in Table S1 in the supplementary information. The CFRTPCs were prepared by a hot-pressing process, and a schematic illustration is shown in Figure 1a. First, the prepreg was stacked and welded by ultrasonic spot welding with a layup sequence of [0/90]_7s_ (i.e., the total thickness was 4.2 mm with 24 plies). Second, the welded preform was placed in a combination die for hot pressing and cured by the process shown in Figure 1b. The die was heated to 360 °C and held at this temperature for 30 min with a pressure of 5 MPa in a hot press. After that, the die was cooled to 140 °C with a pressure of 5 MPa. The CFRTPCs named CF/PAEK were obtained after demolding (as shown in Figure 1c). Finally, the internal quality of the CF/PAEK (see Figure 1d) was characterized using a scanning acoustic microscope (SAM, PVA Tepla SAM 300, Wettenberg, Germany), and the cross section (see Figure 1e) was observed by an optical microscope (Leica DMC 4500, Weztlar, Germany). The internal quality of the CF/PAEK was uniform without obvious defects, and the fiber volume fraction was approximately 53.4%.

### 2.2. Laser Ablation Test

**Figure 2:**
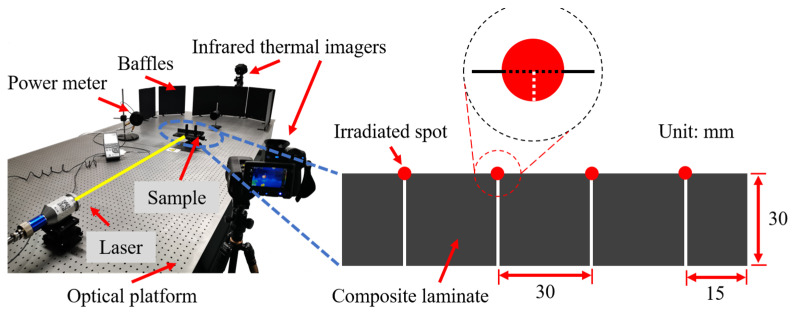
Laser irradiation test platform.

The laser ablation experimental system is shown in Figure 2. The test system was mainly composed of a fiber laser (RFL-C3000S, wavelength 1080 nm, Wuhan Raycus Fibre Laser Technologies Co., Ltd., Wuhan, China), a power meter (PM3K+FieldMaxII-To, Coherent, Palo Alto, CA, USA), baffles, and an optical platform. The CF/PAEK was cut into rectangular samples with a size of 120 mm × 30 mm × 4.2 mm. The spot center was set on the edge of each region to intuitively observe the damage status (see Figure 2). The influence of the laser parameters on the ablation damage of CF/PAEK was studied by changing laser power, irradiation time, and spot diameter. Table 1 lists the 10 irradiation conditions, i.e., No. 1–No. 4, to discuss the effect of laser power, No. 4–No. 7 to investigate the effect of irradiation time, and No. 4, No. 8–No. 10 to study the effect of spot diameter. As shown in Table 1, all three test schemes were controlled by only one variable. The laser power was set from 600 W to 1500 W, the irradiation time was set from 2 s to 8 s, and the spot diameter was set from 1 mm to 4 mm. The weight of the samples before and after the ablation test was measured by an analytical balance with 0.1 mg readability (METTLER TOLEDO, Zurich, Switzerland). After testing, the damaged areas of the ablated composites were characterized by SAM and measured by Photoshop software. The damaged depths of the ablated composites were characterized by a digital microscope (OLYMPUS DSX1000, Tokyo, Japan). The micromorphology of the ablated composites was characterized by scanning electron microscopy (SEM, HITACHI S4800, Tokyo, Japan). The chemical composition of the ablated regions was identified by energy-dispersive X-ray spectroscopy (EDS).

## 3. Results and Discussion

### 3.1. Ablation Performance

**Figure 3:**
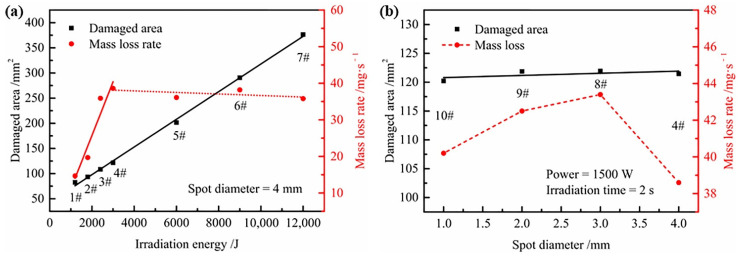
(**a**) Influence of laser energy on damaged area and mass loss rate of CF/PAEK, with the same spot diameter (4 mm); (**b**) influence of the spot diameter on damaged area and mass loss rate of CF/PAEK, with the same laser energy (3000 J).

The changes in laser power (samples 1–4), irradiation time (samples 4–7), and spot diameter (samples 4, 8–10) had different effects on the damage of CF/PAEK, as shown in Table 1. Damaged areas of CF/PAEK characterized by the SAM and damaged depths of CF/PAEK characterized by the digital microscope are respectively presented in Figures S1 and S2 in the Supplementary Information. When the spot diameter was 4 mm (samples 1–7), the relationship between the damaged area and mass loss rate of CF/PAEK on one hand and the irradiation energy on the other is shown in Figure 3a. The damaged area showed an approximately linear relationship with irradiation energy. Since samples 1–4 and samples 4–7 were irradiated at different laser powers and irradiation times, the effects of laser power and irradiation time were equivalent. On the other hand, the damage area did not seem related to the power density, since the power densities of samples 4–7 were constant, while the damage area increased with increasing irradiation energy. Figure 3a also shows that the mass loss rate increased nearly linearly for samples 1–4 and remained constant for samples 4–7. This indicates that the mass loss rate is positively correlated with laser power but not with irradiation energy, because the power densities of samples 1–4 increased linearly, while the power densities of samples 4–7 remained constant. These results are rarely reported in previous works. Since the mass loss rate is primarily caused by ablation, while the damaged area is mainly induced by thermal conduction, the damaged area is controlled by irradiation time and laser power, according to the thermal conduction theory [27]. However, the mass ablation rate pertains to the power density based on the ablation theory proposed by Dimitrienko [28]. Consequently, the damaged area is determined by the irradiation energy, while the mass loss rate is controlled by the laser power density. It should be noted that these linear relationships are only empirical relationships within this energy range. In fact, these relationships cannot be strictly linear due to the influence of air scattering, material energy absorption rate, material phase change, heat transfer, etc.

The relationship between damaged area and mass loss rate of CF/PAEK on one hand and spot diameter on the other is shown in Figure 3b. The damaged area remained almost constant as the spot diameter increased. This further proved that the damage area is related to the irradiation energy but not to the power density. The result is similar to that of Pagano et al. [29]. When the CW laser beam with the same power density passed through the composite, the change of the ablation width with energy density was not obvious. On the contrary, the ablation depth increased significantly with the increase of energy density. However, the mass loss rate increased first and then decreased as the spot diameter enlarged. The power density is inversely proportional to the square of the spot diameter when the laser power and irradiation time are constant. When the spot diameter increases to a certain extent, the laser is insufficient to cause ablation of the material due to the low power density. According to the research by Allheily et al. [4], the surface temperature of composites irradiated by a CW laser increased with the increase of power density. Therefore, a peak of mass loss rate appeared with increasing spot diameter. This indicates that increasing the power density to ablate the composite in the depth direction more easily threatens the internal structure.

In summary, the ablation damaged area is controlled by the irradiation energy, while the mass loss rate is controlled by the power density. According to the above conclusions, different damage effects can be achieved by adjusting the laser parameters. For example, the smallest spot diameter and highest laser power should be chosen to destroy internal electronic components and equipment. In contrast, a high laser power and large spot diameter are more suitable for structural destruction owing to the larger damaged area.

### 3.2. Morphology and Characteristics

**Figure 4:**
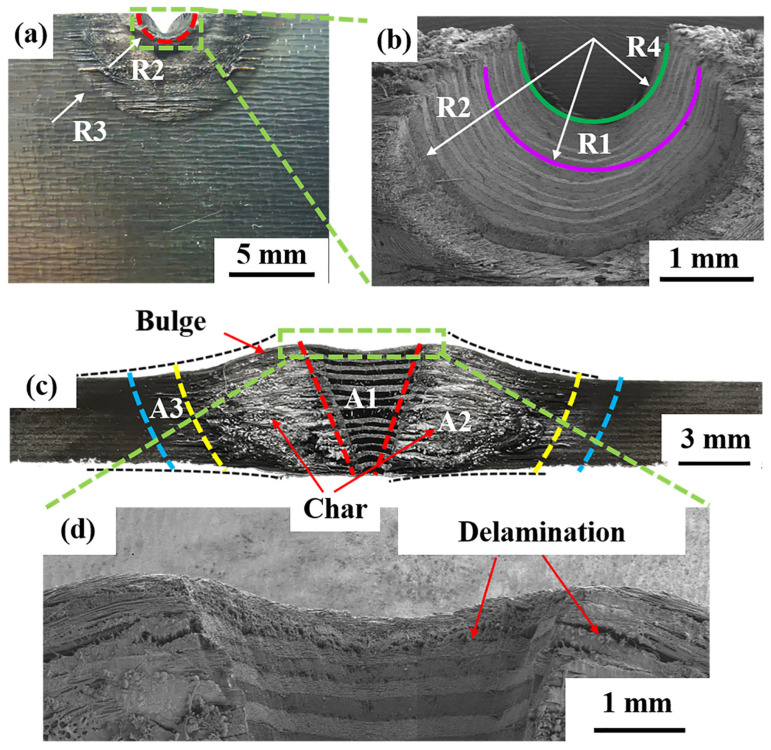
Morphology of the CF/PAEK surface after the laser ablation test: (**a**,**b**) front surface; (**c**,**d**) cross section.

Figure 4 shows the typical morphology of a specimen after laser ablation. As shown in Figure 4a, there are three distinct semicircles in the front view of the ablation region, which can be divided into an ablation center (inner red circle, labelled R2), a transition zone (between the yellow and red circles), and a heat-affected zone (between the yellow and blue circles, labelled R3). Furthermore, the original spot radius R1 was larger than the bottom radius (i.e., R4) of the ablation center but smaller than the top radius (i.e., R2) of the ablation center (see Figure 4b). This result denoted that also outside of the laser beam, resin and fiber decomposed and sublimated due to the high temperature. The above phenomena are similar to those observed for thermoset composites, in which the diameter of the ablation zone was larger than that of the laser spot, and the energy of the laser damaged the vicinity of the ablation center through heat conduction [10,11,15,30]. Because the temperature in the direct laser irradiation region was extremely high, a high-temperature zone also formed in the surrounding area due to heat conduction and convection, which caused the PAEK resin to fuse and the CF to sublimate, resulting in a significantly larger damage projection diameter of the upper surface with respect to that of the spot. In the ablation center, the PAEK resin and CF were ablated, leaving an ablation pit. In the transition zone, the PAEK resin experienced decomposition, fusion, and cooling processes. In the heat-affected zone, there were only fusion and cooling processes (see Figure 4a). These phenomena indicate that decomposition and fusion phenomena were closely related to the temperature distribution. In addition, there was no obvious ablation in the CFs in these two zones because of the relatively low temperature. However, in the ablation center of the thermoset composite, the resin and carbon fibers were decomposed or sublimated directly, and there was no clear transition zone [9,10]. These differences will greatly affect the residual strength of the composite after laser ablation.

Figure 4c shows that the shape of the ablation center looked like a cone. This was because the energy distribution of the laser was Gaussian, and there was no significant change in the energy distribution of the laser during ablation. Therefore, the specimen formed an inverted conical ablation pit along the laser propagation direction with limited irradiation time [31,32]. Similarly, the ablation center, transition zone, and heat-affected zone can also be clearly distinguished from the cross-section morphology (see Figure 4c). In addition, there was plenty of residual carbon attached to the surface of the transition zone. Furthermore, massive delamination caused by thermal stress can be observed in the cross section of the ablation center and transition zone (see Figure 4c,d). The gas pressure caused by resin decomposition was another reason for delamination. However, no delamination could be found in the heat-affected zone, since the PAEK resin only remelted instead of decomposing, which is significantly different from what observed for a thermoset resin, where the boundary of the ablation center was not clear, and there was an obvious hole structure 10. Therefore, the delamination area of CF/PAEK may be smaller than that of a thermoset composite and maintain a higher damage tolerance. This result is crucial for the laser damage assessment of thermoplastic composites.

**Figure 5:**
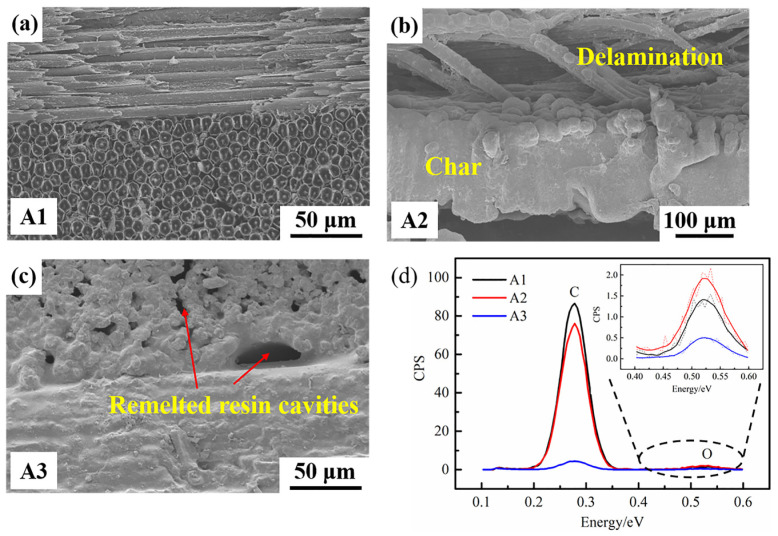
Micromorphology of the CF/PAEK cross section after the laser ablation test: (**a**) region A1; (**b**) region A2; (**c**) region A3; (**d**) EDS pattern of regions A1, A2, and A3.

Figure 5 shows the lateral micromorphology (i.e., regions A1, A2, and A3 in Figure 4c) and element composition of the ablated region. It is clear that two orthogonally oriented broken CFs can be found in the ablation pit (as shown in Figure 5a). The damage effect of the transition zone was gradient-distributed; the closer to the ablation center, the more serious the damage, which is consistent with the phenomena observed in the front view of the ablation region. Meanwhile, a large quantity of resin decomposition and carbonization products could be observed on the surface of the transition zone. Furthermore, Figure 5b shows that the resin among the fibers was completely carbonized, and obvious delamination was found in the transition zone. This was probably caused by thermal stress and gas generated from the resin decomposition. In contrast, the heat-affected zone was less affected by high temperature, and the PAEK resin was retained among the fibers. However, many cavities formed after resin fusion and cooling (see Figure 5c). These phenomena are different from those observed in thermoset composites, where there was no obvious division around the ablation pit, and the edges were blurred with only fibers 91,014. The carbon/oxygen atomic ratio obtained by EDS is shown in Figure 5d. The carbon/oxygen atomic ratios of A1 and A2 were as high as 61:1 and 40:1, respectively, which was caused by the decomposition and carbonization products of CF and PAEK resin after ablation. In contrast, the carbon/oxygen atomic ratio of A3 was only 9:1, which corresponded to the remelted PAEK resin.

### 3.3. Ablation Evolution Behavior of Fibres

**Figure 6:**
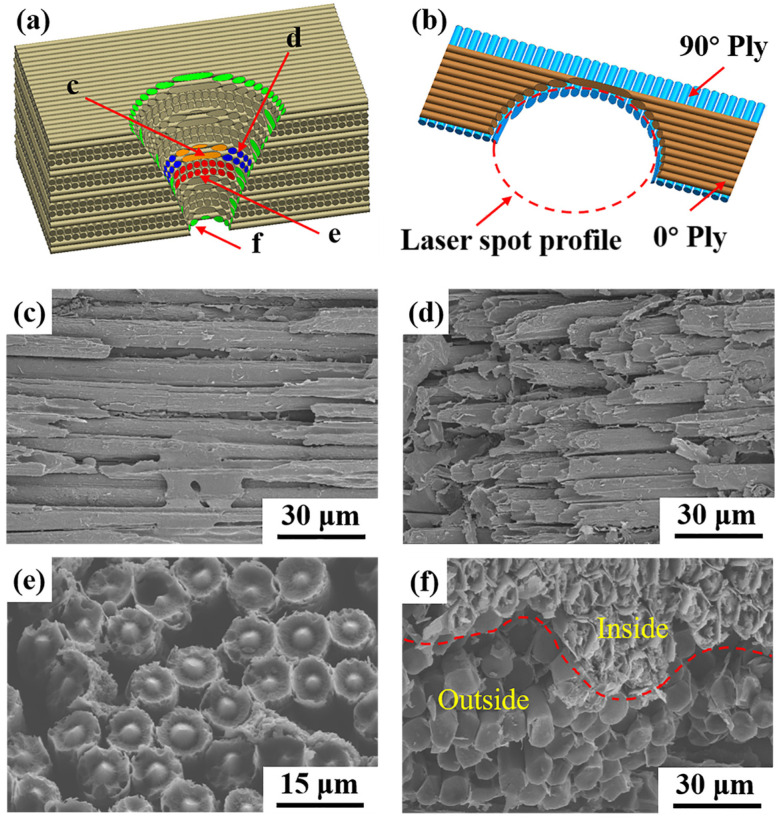
Ablation morphology of CFs in the ablation pit: (**a**) schematic for layup; (**b**) schematic for 0°/90° plies; SEM photograph for (**c**) center of 0° ply; (**d**) edge of 0° ply; (**e**) 90° ply; (**f**) edge of the ablation pit.

The ablation behavior and its mechanism are significant issues in laser damage assessment. Figure 6 shows that four obviously different ablation morphologies of CF were observed in the ablation pit, which were located in different positions. When the laser beam irradiated the surface of CF/PAEK, the PAEK resin decomposed first due to the extremely rapid heating process. As the resin decomposed into gas, a relatively positive pressure environment formed in this region, resulting in air being inaccessible to this zone. Therefore, resin and CF decomposed and sublimated in an anoxic environment. In addition, the high-temperature gas generated by the decomposition of PAEK resin and the sublimation of CF continuously absorbed heat and eroded the ablation pit, causing decomposition of the PAEK resin and sublimation of the CF outside the laser spot. Hence, the diameter of the ablation pit (i.e., R2 in Figure 4b) became larger than that of the laser spot (i.e., R1 in Figure 4b).

As shown in Figure 6b, 0° plies and 90° plies showed different ablation morphologies when they were cut by the circular section of the laser spot. The 0° plies showed two different ablation morphologies, depending on the geometric relationship between the laser spot and the plies. The front of the laser spot circular section was tangent to the 0° plies; thus, the remaining 0° fibers were not cut off but were connected in filaments on both sides, as shown in Figure 6c. Meanwhile, the side of the laser spot circular section cut 0° fiber bundles, forming a multilayer stacked morphology, as shown in Figure 6d. Moreover, the ablated 90° fibers presented more significant characteristics, as shown in Figure 6e,f. The fiber section in the ablation pit was needle-like, and the outside was surrounded by a circle of loose cortex, forming a cylindrical nested cone structure, as shown in Figure 6e. However, the fiber section around the ablation pit was a smooth circle or oval, as shown in Figure 6f. This phenomenon was rarely reported in previous laser ablation behavior studies of resin matrix composites [30,33]. However, this phenomenon and its mechanism are significant to laser machining and laser damage evaluation.

During the preparation of CF, a slight cortex–core structure of the precursor fiber formed due to double diffusion in the coagulation bath, which further intensified during the subsequent carbonization. The cortex was densely arranged and aligned along the axial direction with fewer defects, while the core was disordered, with a large number of pores [34,35,36]. Therefore, the heterogeneous structure along the radial direction of CF led to an uneven distribution of the thermophysical parameters. Specifically, the core should have the largest specific heat and the lowest thermal conductivity, while the cortex should have opposite values. It is noteworthy that the thermophysical properties of materials significantly influence the ablation process and morphology. Since the decomposition temperature of PAEK (i.e., 590 °C) is far lower than the sublimation temperature of CF (i.e., 3550 °C), the axial thermal conductivity of CF is much higher than that of PAEK, and the ablation rate of PAEK is much larger than that of CF. On the other hand, the highest specific heat of the core led to the lowest temperature rise, resulting in the earliest sublimation of the cortex. Consequently, the needle-like structure was formed instantaneously. Liu et al. [37] also reported this needle-like morphology of CF. The reason of this morphology in that paper was that the core part of CF reached the highest decomposition temperature. There are some other similar reports about the needle-like morphology of CF by Chen et al. [38] and Li et al. [39]. However, a cylindrical nested cone structure was never reported, probably because the matrix in previous research was SiC. Due to the anoxic environment in the ablation pit, PAEK resin decomposition left residual carbon, and only approximately 40% of the weight was lost when the temperature reached the carbonization temperature [40]. Therefore, a thin layer of residual carbon would leave the outside of CFs, forming a cylindrical nested cone structure. However, in the aerobic environment at the edge of the ablation pit, the PAEK resin could be oxidized, and there was no residual carbon layer. In addition, the fiber could be continuously oxidized at a temperature lower than the sublimation point [41,42]; thus, a flat section finally formed.

### 3.4. Ablation Mechanism

**Figure 7:**
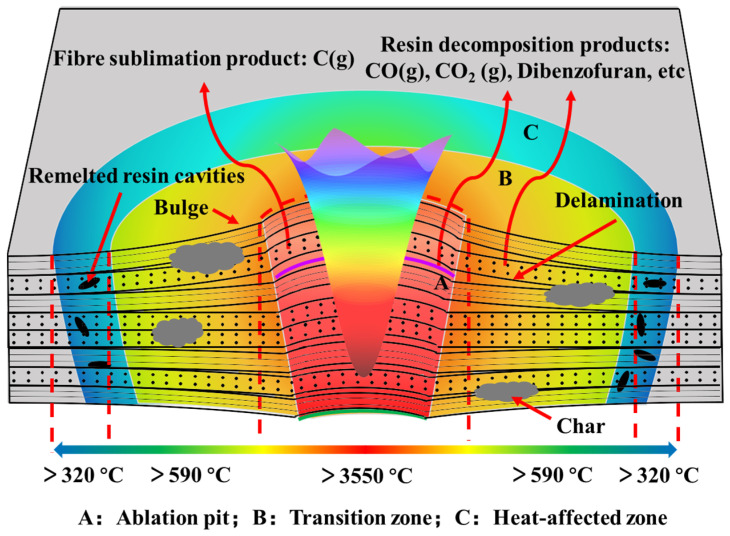
Schematic of the ablation mechanism of CF/PAEK.

According to the above analysis, the ablation mechanism of CF/PAEK under CW laser irradiation can be summarized as follows (see Figure 7).

When a high-energy CW laser irradiates the surface of the CF/PAEK laminate, the temperature in the spot center increases rapidly, causing the PAEK resin and the CFs to decompose and sublimate instantly. According to the temperature distribution, the ablation area can be divided into three regions: ablation center, transition zone, and heat-affected zone. The ablation center is directly irradiated by the laser, showing the fastest heating rate and the highest temperature (i.e., >3550 °C). The PAEK resin first decomposes to produce small molecular gaseous products. When the temperature continues to rise above the sublimation temperature of the CF, the CFs sublimate into gas. Since the laser energy has a Gaussian distribution, a V-shaped ablation pit is formed. Due to the rapid formation of abundant gaseous products, a positive vapor pressure is formed, which prevents external air from entering the ablation pit. Therefore, the PAEK resin decomposes and leaves a thin residual carbon layer on the surface of CFs. Meanwhile, the CFs sublimates and forms a needle-like structure owing to the inhomogeneity of the fibers along the radial direction. Therefore, the unique cylinder nested cone structure of CF forms.

The temperature in the transition region is between the decomposition temperature of the PAEK resin and the sublimation temperature of CF. In the presence of air, the PAEK resin and the CFs on the surface layer are oxidized to gaseous products such as CO and CO_2_ [43]. In contrast, in the internal anaerobic environment, the PAEK resin decomposes into gaseous products, and residual carbon is formed and deposited on the surface of the composite at high temperature. Thus, interlaminar delamination also occurs in the composite. Therefore, the damage mechanism of the transition zone is thermochemical oxidation and decomposition of the PAEK resin and thermochemical oxidation of the CF. In addition, the lack of interlaminar resin leads to the delamination of the composite in the ablation pit, and the top and bottom surfaces bulge outward.

The temperature in the heat-affected zone is between the melting temperature and the decomposition temperature of the PAEK resin. The PAEK resin undergoes physical changes associated with remelting and solidification during heating and cooling, while the CFs do not change in this temperature range. The PAEK resin flows locally under gravity in the molten state, leaving cavities in several areas after solidification. Since most resin remains in the interlaminate, and no gas decomposition product is generated, there is no delamination in the heat-affected zone. Therefore, the damage mechanism in this region is only thermophysical melting of the PAEK resin, which is different from that observed for the thermoset resin.

The above mechanism shows that the ablation behavior of thermoplastic composites is obviously different from that of thermosetting composites in some respects. Compared with thermoset resins, CF/PAEK formed a molten layer during the laser ablation process, which protected the material from further oxidation and damage. This property enables the composite structures to maintain high residual strength after laser ablation, thus showing good laser damage resistance.

## 4. Conclusions

This paper concentrated on the ablation mechanism and performance of CF/PAEK thermoplastic composites subjected to CW laser irradiation. The effects of laser power, irradiation time, and spot diameter on the ablation damaged area and mass loss rate of CF/PAEK thermoplastic composites were discussed. The results showed that the ablation damaged area is controlled by the irradiation energy, while the mass loss rate is controlled by the power density. The morphology and composition of the ablated composite were characterized to reveal the ablation mechanism. According to the temperature distribution, the ablation area was divided into three regions: ablation center, transition zone, and heat-affected zone. The formation mechanism of the unique cylinder nested cone ablation morphology of CF was explained.

The ablation mechanism presented in this study can deepen our understanding of the damage evolution of CF/PAEK subjected to high-energy CW laser irradiation. Moreover, this work lays the foundation for subsequent research on CF/PAEK damage tolerance after laser ablation. The residual strength of the damaged laminate can be tested, and the strain response of the laminate during laser ablation can be tested in situ. In addition, research about the development of laser ablation-resistant coatings and protective effects is meaningful.

## Figures and Tables

**Table 1:** Experimental parameters of the laser ablation test and experimental results.

Number	LP/W	IT/s	SD/mm	MLR/mg·s^−1^	DA/mm^2^
1	600	2	4	14.7	82.4
2	900	2	4	19.7	93.4
3	1200	2	4	35.9	118.4
4	1500	2	4	38.6	121.4
5	1500	4	4	36.1	201.4
6	1500	6	4	38.2	290.4
7	1500	8	4	35.8	376.4
8	1500	2	3	43.4	121.9
9	1500	2	2	42.5	121.9
10	1500	2	1	40.2	120.2

*Note*: LP is laser power; IT is irradiation time; SD is spot diameter; MLR is mass loss rate (mass loss per second); DA is damaged area.

## Data Availability

Data in this paper has not been reported.

## Supplementary Materials

The following supporting information can be downloaded at: https://www.mdpi.com/article/10.3390/polym14132676/s1, Table S1: Thermophysical properties of CF and PAEK-L resin, Figure S1: Damaged areas of CF/PAEK characterized by the SAM, Figure S2: Damaged depths of CF/PAEK characterized by digital microscopy.
