# Supplementary material for: Laser Ablation Mechanism and Performance of Carbon Fiber-Reinforced Poly Aryl Ether Ketone (PAEK) Composites
Source: Polymers (Basel). 2022 Jun 30;14(13):2676. doi: 10.3390/polym14132676 (PMC9269289; doi:10.3390/polym14132676)
Supplement: Supplementary file 1 [file polymers-14-02676-s001.zip › polymers-1782371-supplementary.pdf]

Electronic Supplementary Information (ESI):

Laser ablation mechanism and performance of carbon fibre reinforced poly aryl ether ketone (PAEK) composites

Jindong Zhang [1], Ran Bi [2], Shengda Jiang [2], Zihao Wen [2], Chuyang Luo [2,*], Jianan Yao [1], Gang Liu [1,*], Chunhai Chen [1,2] and Ming Wang [1]

[1] Center for Advanced Low-Dimension Materials, State Key Laboratory for Modification of Chemical Fibers and Polymer Materials, College of Materials Science and Engineering, Donghua University, Shanghai 201620, China; zhangjindong@buaa.edu.cn (J.Z.); yjn@dhu.edu.cn (J.Y.); cch@dhu.edu.cn (C.C.); mwang@dhu.edu.cn (M.W.)

[2] Shanghai High Performance Fibers and Composites Center (Province-Ministry Joint), Center for Civil Aviation Composites, Donghua University, Shanghai 201620, China; 2210510@mail.dhu.edu.cn (R.B.); jiangshengda321@163.com (S.J.); 15221820839@163.com (Z.W.)

* Correspondence: cyluo@dhu.edu.cn (C.L.); liugang@dhu.edu.cn (G.L.)

List of Contents for Supplementary Materials:

Table S1 Thermophysical properties of CF and PAEK-L resin.

Figure S1 Damaged areas of CF/PAEK characterized by the SAM.

Figure S2 Damaged depths of CF/PAEK characterized by digital microscopy.

Table S1 Thermophysical properties of CF and PAEK-L resin.

Parameters	CF*	PAEK-L**
Density /g·cm^{-3}	1.80	1.29
Specific heat /J·g^{-1}·K^{-1} (25 °C)	0.75	1.05
Thermal conductivity /W·m^{-1}·K^{-1} (25 °C)	k_{11} = 9.38	k = 0.24
Melting temperature /°C	/	284-327
Sublimation point /°C	3550	/

Note: *Provided by Weihai Tuozhan Fibre Co., Ltd.

**Provided by Heilongjiang Yingchuang New Materials Co., Ltd.

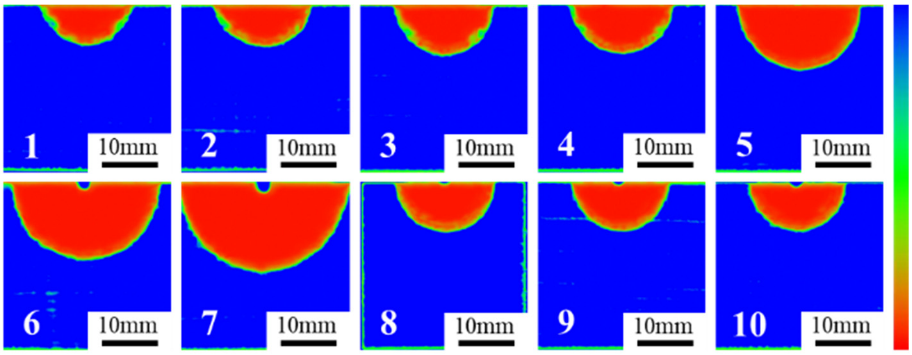

Figure S1 Damaged areas of CF/PAEK characterized by the SAM.

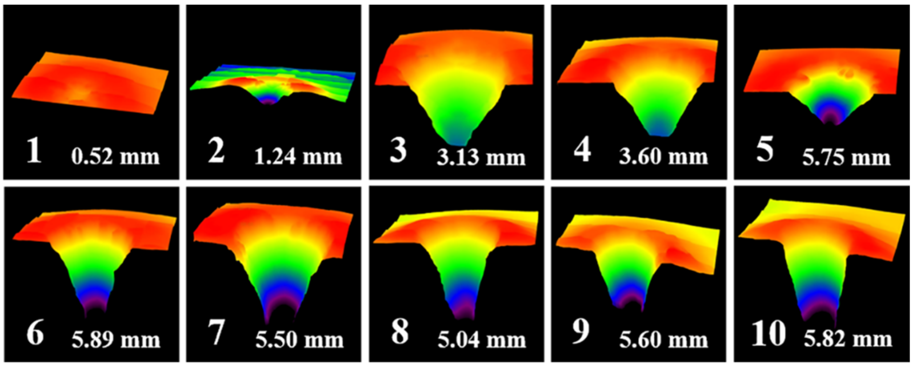

Figure S2 Damaged depths of CF/PAEK characterized by the digital microscope.
